# Discovering genetic linkage between periodontitis and type 1 diabetes: A bioinformatics study

**DOI:** 10.3389/fgene.2023.1147819

**Published:** 2023-03-27

**Authors:** Junqi Liu, Bo Zhang, Guanyin Zhu, Chenlu Liu, Shuangcheng Wang, Zhihe Zhao

**Affiliations:** ^1^ State Key Laboratory of Oral Diseases and National Clinical Research Center for Oral Diseases, West China Hospital of Stomatology, Sichuan University, Chengdu, China; ^2^ Department of Orthodontics, West China Hospital of Stomatology, Sichuan University, Chengdu, China

**Keywords:** periodontitis, type 1 diabetes, genetic linkage, hyperglycaemia, bioinformatics

## Abstract

**Background:** Relationship between periodontitis (PD) and type 1 diabetes (T1D) has been reported, but the detailed pathogenesis requires further elucidation. This study aimed to reveal the genetic linkage between PD and T1D through bioinformatics analysis, thereby providing novel insights into scientific research and clinical treatment of the two diseases.

**Methods:** PD-related datasets (GSE10334, GSE16134, GSE23586) and T1D-related datasets(GSE162689)were downloaded from NCBI Gene Expression Omnibus (GEO). Following batch correction and merging of PD-related datasets as one cohort, differential expression analysis was performed (adjusted *p*-value <0.05 and ∣log_2 _ fold change| > 0.5), and common differentially expressed genes (DEGs) between PD and T1D were extracted. Functional enrichment analysis was conducted *via* Metascape website. The protein-protein interaction (PPI) network of common DEGs was generated in The Search Tool for the Retrieval of Interacting Genes/Proteins (STRING) database. Hub genes were selected by Cytoscape software and validated by receiver operating characteristic (ROC) curve analysis.

**Results:** 59 common DEGs of PD and T1D were identified. Among these DEGs, 23 genes were commonly upregulated, and 36 genes were commonly downregulated in both PD- and T1D-related cohorts. Functional enrichment analysis indicated that common DEGs were mainly enriched in tube morphogenesis, supramolecular fiber organization, 9 + 0 non-motile cilium, plasma membrane bounded cell projection assembly, glomerulus development, enzyme-linked receptor protein signaling pathway, endochondral bone morphogenesis, positive regulation of kinase activity, cell projection membrane and regulation of lipid metabolic process. After PPI construction and modules selection, 6 hub genes (CD34, EGR1, BBS7, FMOD, IGF2, TXN) were screened out and expected to be critical in linking PD and T1D. ROC analysis showed that the AUC values of hub genes were all greater than 70% in PD-related cohort and greater than 60% in T1D-related datasets.

**Conclusion:** Shared molecular mechanisms between PD and T1D were revealed in this study, and 6 hub genes were identified as potential targets in treating PD and T1D.

## 1 Background

Diabetes mellitus (DM) is a metabolic disorder featured by hyperglycemia as a result of deficiency in insulin secretion and/or insulin action (Rayburn, 1997/9). The incidence of DM is estimated to rise to 629 million by 2045, leading to high public health burden (Simeni Njonnou et al., 2020). DM is associated with a number of devastating complications, which are the leading cause of morbidity and mortality in diabetic individuals. Among all diabetic complications, PD has recently ignited great interest due to its close relation with systemic health. As the sixth complication of DM, PD referrers to a chronic inflammatory disease of periodontal tissues featured by progressive destruction of tooth-supporting structures (Papapanou et al., 2018). PD has influenced at least 40% of adults in America and is the major cause of tooth loosening in adults, exerting negative impact on quality of life (Eke et al., 2000).

PD and hyperglycemia are closely connected. On the one hand, hyperglycemia can significantly increase the susceptibility and severity of PD (Mealey and Ocampo, 2000). On the other hand, PD can impede glycemic control and increase susceptibility to other DM-related complications (Graziani et al., 2018; Taylor et al., 2013; Emrich et al., 1991). In addition, clinical evidence has indicated that effective treatments of PD or desirable glycemic control could alleviate clinical symptoms of the other disease (Madianos and Koromantzos, 2018; Engebretson and Kocher, 2013; Sundar et al., 2018/12; Tsai et al., 2002; Preshaw et al., 2012).

Type 1 diabetes (T1D) is a subtype of insulin-dependent DM caused by destruction of the pancreatic islets. The incidence of T1D has been increasing by 2%–5% globally in the past decades, imposing increasingly heavy burden to healthcare providers (Maahs et al., 2010; Kakleas et al., 2015). Due to a lack of diagnostic biomarkers, the long-term survival rate of T1D is quite low (Pujar et al., 2022). T1D shares several similar clinical symptoms with type 2 diabetes (T2D), such as hyperglycemia and hyperglycemia-related complications like PD. Extensive studies have shown that periodontal parameters were positively correlated with T1D, including gingival index, bleeding on probing, the amount of dental plaque and probing pocket depth (Dakovic and Pavlovic, 2008; Orbak et al., 2008; Popławska-Kita et al., 2014; Ismail et al., 2015; Jindal et al., 2015; Roy et al., 2019; Dicembrini et al., 2020). Radiographic examination also showed that T1D patients presented exacerbated alveolar bone loss compared with non-diabetic individuals (Plessas et al., 2018). In addition, T1D patients were reported to have lower rate of salivary flow and reduced salivary pH, which could promote the formation of dental plaque and further deteriorate the periodontal health (Aren et al., 2003; Hodge et al., 2012). A recent meta-analysis also indicated a strong link between PD and T1D (Rapone et al., 2020a).

Given to the different pathogenic mechanisms, the two types of DM are considered as distinct diseases clinically. Unlike T2D, which primarily affects adults, T1D usually starts early in life and increases the risk of periodontal destruction since childhood and adolescence (Lalla et al., 2007). Although both T1D and T2D have a close association with PD, but clinical studies showed the glycemic control after periodontal treatment is different between T1D and T2D patients, indicating that the underlying genetic linkage between PD and the two types of DM is not exactly the same (Rapone et al., 2020a; Janket et al., 2005/12; Reddy and Gopalkrishna, 2022; Shinjo et al., 2019; Pranckeviciene et al., 2017; Novotna et al., 2015; Rapone et al., 2020b; Corbella et al., 2013). Currently, extensive studies have explored the association between T2D and PD, while there is a paucity of reports regarding deep research on crosstalk between T1D and PD.

Considering the relationship between PD and T1D, investigating genetic linkage between them and uncovering the critical genes are of urgent need and can provide important insights into scientific researches and clinical treatments of the two diseases. Previous studies have indicated that several potential molecules and signaling pathways were pertinent to interplay between PD and T1D (Popławska-Kita et al., 2014; Rapone et al., 2020a). However, genetic crosstalk between T1D and PD is far more complicated, making it difficult to comprehensively elucidate the complex mechanisms only by clinical and experimental studies alone.

Nowadays, bioinformatics analysis is a critical tool in mining novel biomarkers and genetic linkages of diseases. Crosstalk genes and molecular processes can be uncovered and comprehensively described through integrating and analyzing the transcriptomic data. Especially in the era of precise medicine, uncovering the critical disease biomarkers serves as a robust tool to improve disease diagnosis, develop targeted therapies, and predict disease prognosis. In this study, gene expression profiling data of chronic PD and T1D were obtained from four publicly available datasets. After a series of bioinformatic analysis processes, potential molecular mechanisms linking T1D and PD and common dysregulated genes between the two diseases were identified. Finally, critical crosstalk genes were further screened out, potentially providing novel insights into targeted therapeutic strategies for PD and T1D.

## 2 Methods

### 2.1 Datasets acquisition and preparation

Expression data of chronic PD and T1D were downloaded from GEO database (https://www.ncbi.nlm.nih.gov/geo/) (Barrett et al., 2013/1). GSE10344 (Demmer et al., 2008), GSE16134 (Kebschull et al., 2014) and GSE23586 (Abe et al., 2011) were for PD, and GSE162689 was for T1D (Seiron et al., 2021) respectively. The detailed information of these datasets was listed in Table 1.

**TABLE 1:** Description of included datasets.

Datasets	Disease	Type of tissue	Sample		Platform
			Control disease		
GSE10334	PD	Gingival tissue	64	183	Affymetrix human genome U133 plus 2.0 array
GSE16134	PD	Gingival tissue	69	241	Affymetrix human genome U133 plus 2.0 array
GSE23586	PD	Gingival tissue	3	3	Affymetrix human genome U133 plus 2.0 array
GSE162689	T1D	Islets	32	27	Ion torrent S5 XL

For three PD datasets, batch correction was performed using “SVA” package in R language software (R 4.1.0). In this way, the three PD datasets were combined as one cohort with 136 control samples and 427 PD samples. Principal component analysis (PCA) on expression values of samples was conducted before and after batch correction *via* “library (vegan)” package and the PCA results were visualized by “ggplot2” package. The probe ID was converted into genetic symbol according to annotation files. When multiple probes matched one single gene, the gene expression value was calculated by the average level of the probes. Additionally, probes that did not match the genetic symbols were removed.

**FIGURE 1:**
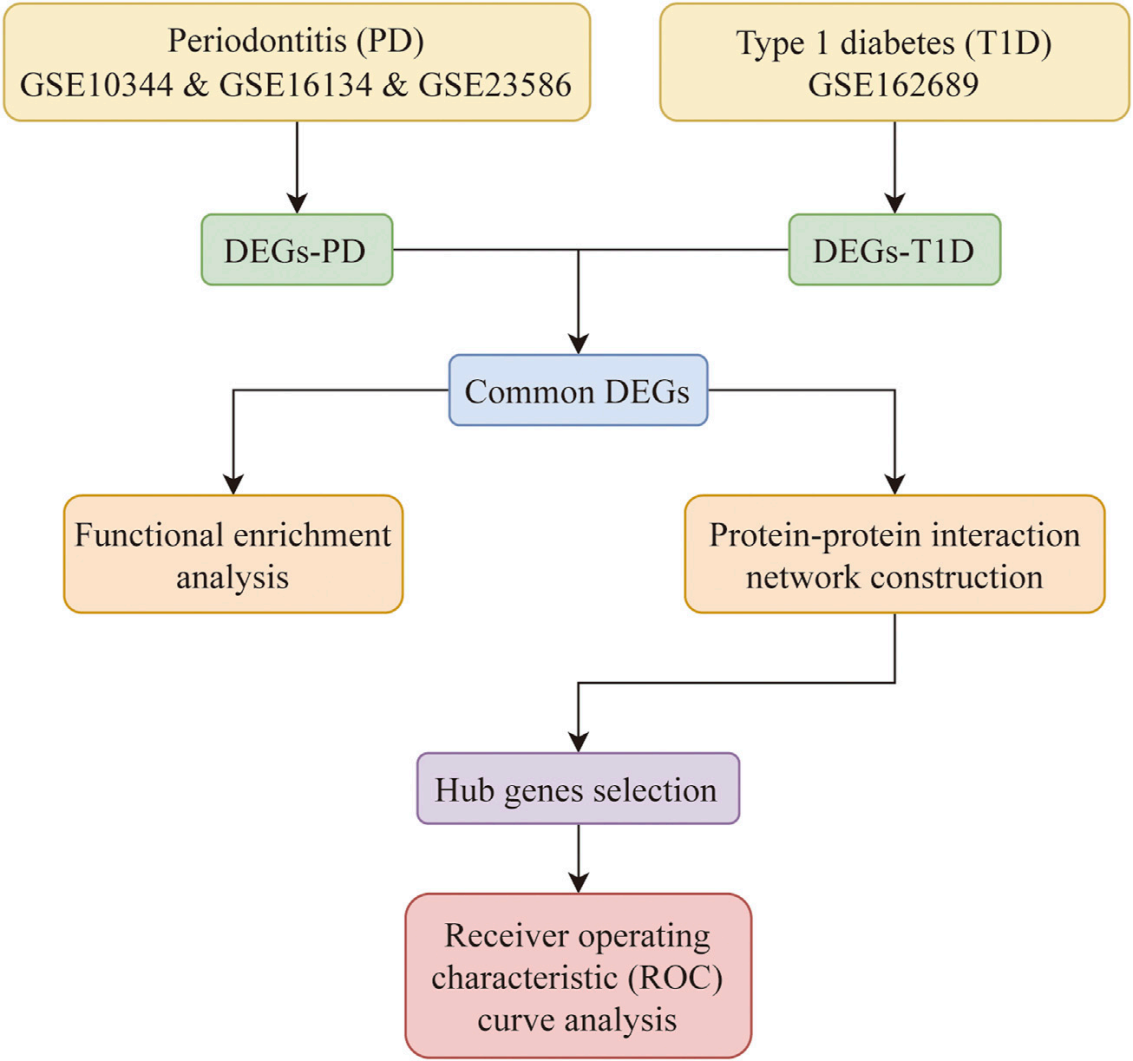
The workflow of this study.

The workflow of this study was presented in Figure 1.

### 2.2 Identification of DEGs

To identify the DEGs between disease and normal samples, the “limma” package and “DESeq2” package in R language software were used for PD- and T1D-related datasets respectively. Adjusted *p*-value <0.05 and |log_2_ fold change| >0.5 was set as the cut-off criteria of DEGs, and log_2_ fold change >0.5 was for upregulated genes and log_2_ fold change <0.5 was for downregulated genes. Subsequently, common upregulated and downregulated DEGs of PD and T1D were obtained by overlapping the two cohorts of DEGs. The “venn” package in R language software was employed to plot Venn diagrams.

### 2.3 Functional enrichment analysis

The functional enrichment analysis of common DEGs was conducted using the webtool “Metascape” (http://metascape.org/) (Zhou et al., 2019). Metascape is up-to-date and contains a broad set of gene list annotation and analysis resource. In this study, the following databases were adopted for pathway and processes enrichment: Gene Ontology (GO) Molecular Functions, GO Biological Processes, GO Cellular Components, Kyoto Encyclopedia of Genes and Genomes (KEGG) Pathway, Reactome Gene Sets, WikiPathways, Canonical Pathways and PANTHER Pathways. Min overlap: 3, *p*-value: 0.05 and Min enrichment: 1.5 were set as cutoff criteria. The significance was ranked by -log_10_ (*p*-value).

### 2.4 PPI network construction and hub genes selection

PPI network was constructed *via* STRING database (Szklarczyk et al., 2019). Further, the PPI network was imported into Cytoscape software for visualization and hub gene selection. The plugin “Cytohubba” in Cytoscape was employed to select key gene modules (Chin et al., 2014). Top 10 highly connected genes of three topological algorithms (Degree, MCC and Stress) were obtained and overlapping genes of the three sets of genes were considered as hub genes. Boxplots of expression value of hub genes was drawn *via* R’s “ggplot2” and “ggpubr” package.

### 2.5 ROC curve analysis

To evaluate the diagnostic performance of hub genes in each disease, “pROC” package in R language software was used to perform ROC curve analysis. Area under the curve (AUC) reflect the prediction effect of the hub genes.

## 3 Results

### 3.1 Identification of common DEGs in PD and T1D

**FIGURE 2:**
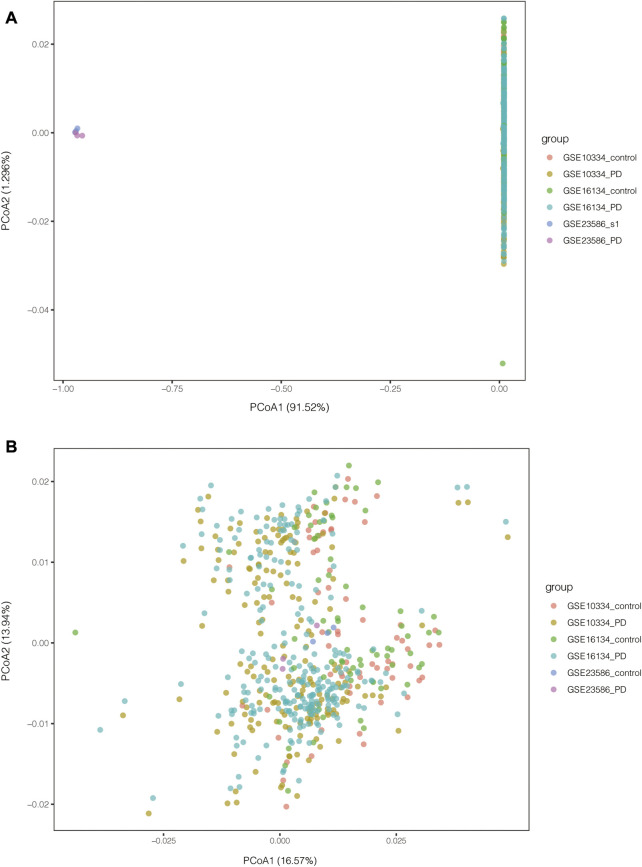
PCA results of PD-related datasets before and after batch correction. **(A)** PCA results before batch correction; **(B)** PCA results after batch correction.

**FIGURE 3:**
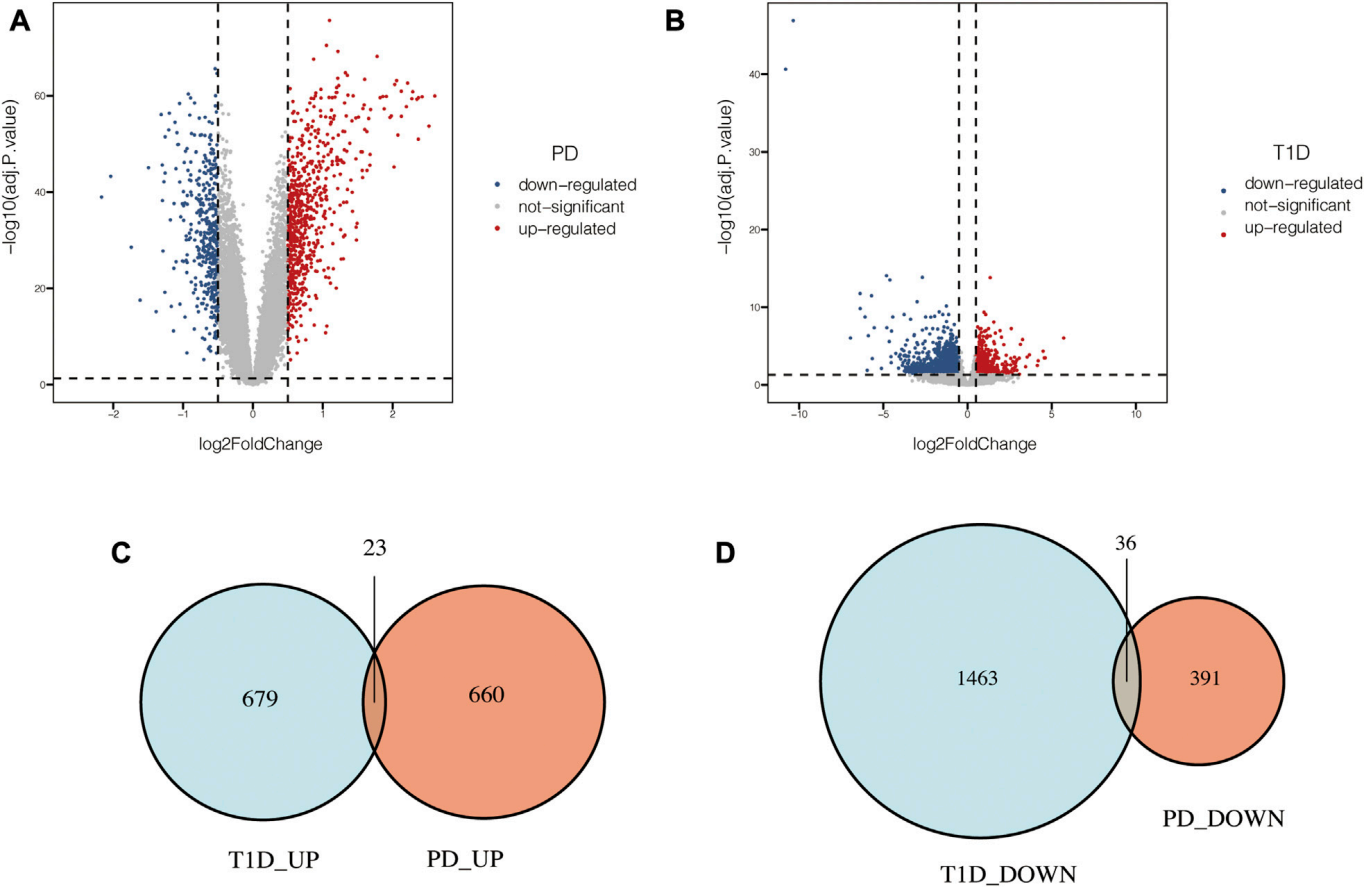
Common DEGs in PD and T1D. **(A)** Volcano plots of DEGs identified in PD datasets. **(B)** Volcano plots of DEGs identified in T1D datasets. **(C)** Venn diagram of upregulated common DEGs. **(D)** Venn diagram of downregulated common DEGs.

PCA of the expression values indicated a successful batch correction (Figure 2). For PD, 1110 DEGs were identified, of which 683 genes were upregulated and 427 genes were downregulated. 2201 DEGs were identified in T1D datasets, of which 702 genes were upregulated and 1499 genes were downregulated (Figures 3A, B). Through taking the intersection of upregulated and downregulated DEGs respectively, 23 upregulated and 36 downregulated common DEGs were screened out, namely, 59 common DEGs were obtained (Figures 3C, D).

### 3.2 Functional enrichment analysis

**FIGURE 4:**
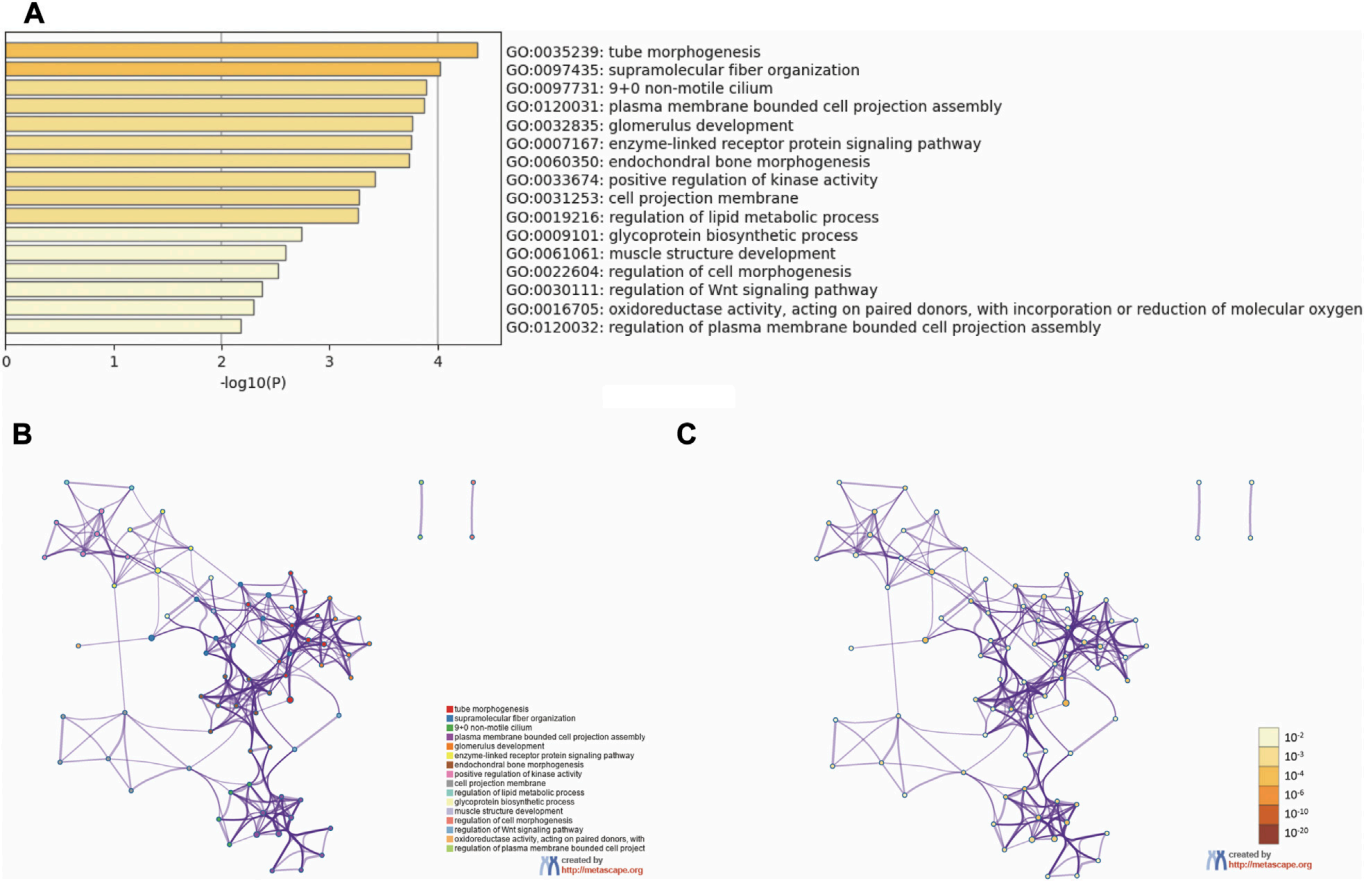
Functional enrichment analysis. **(A)** Bar graph of enriched terms. **(B)** Network of enriched terms colored by cluster ID. **(C)** Network of enriched terms colored by *p*-value.

Functional enrichment analysis was conducted to explore the function of the 59 common DEGs. As the result showed, the common DEGs were enriched in GO Biological Processes and GO Cellular Components (Figure 4). The top 10 terms are tube morphogenesis, supramolecular fiber organization, 9 + 0 non-motile cilium, plasma membrane bounded cell projection assembly, glomerulus development, enzyme-linked receptor protein signaling pathway, endochondral bone morphogenesis, positive regulation of kinase activity, cell projection membrane and regulation of lipid metabolic process.

### 3.3 PPI network construction and hub genes selection

**FIGURE 5:**
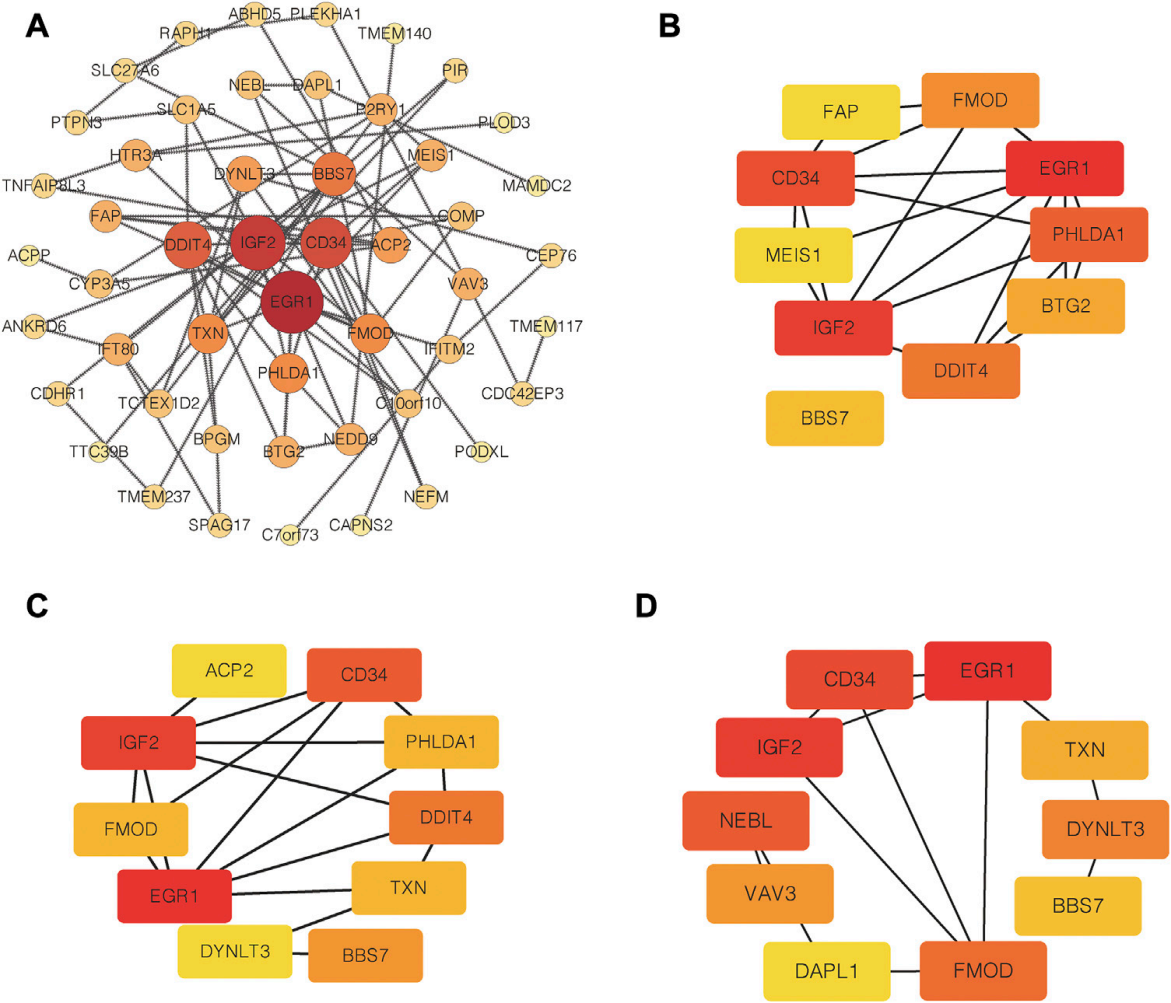
PPI network construction and hub genes selection. **(A)** PPI network of common DEGs. **(B)** Top 10 highly connected genes based on MCC algorithms. **(C)** Top 10 highly connected genes based on degree algorithms. **(D)** Top 10 highly connected genes based on Stress algorithms.

**FIGURE 6:**
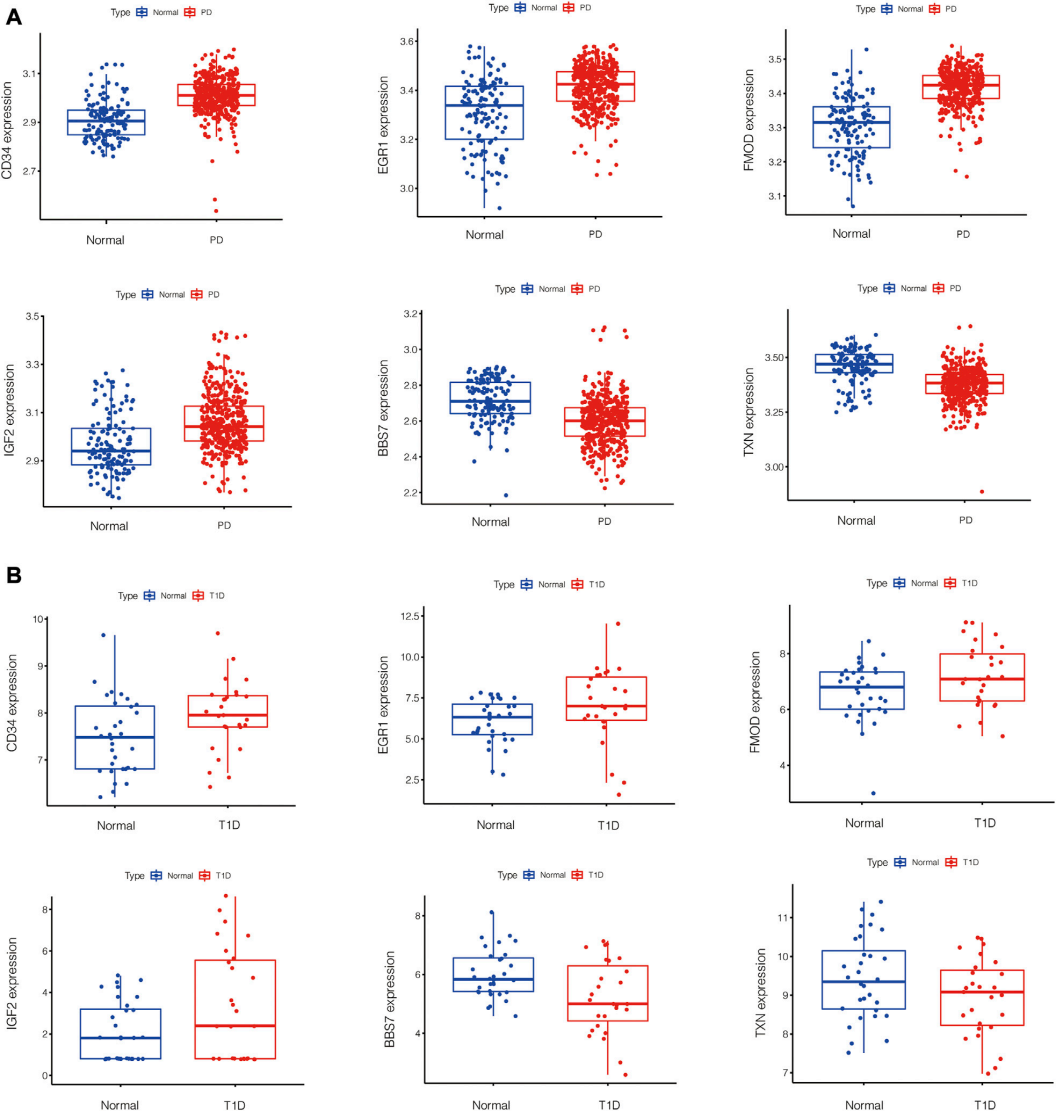
Box plots of hub genes expression in PD **(A)** and T1D **(B)** datasets.

PPI network of common DEGs was shown in Figure 5A. Based on three topological algorithms (MCC, Degree and Stress), three groups of top 10 highly connected genes were obtained (Figures 5B–D), common genes of which were selected as hub genes. 6 hub genes were identified in this study: CD34, EGR1, BBS7, FMOD, IGF2, TXN. The expression value of hub genes in each disease cohort showed that CD34, EGR1, FMOD, IGF2 were upregulated in PD and T1D, while BBS7 and TXN were downregulated in the two diseases (Figure 6).

### 3.4 Identification of diagnostic performance of hub genes

**FIGURE 7:**
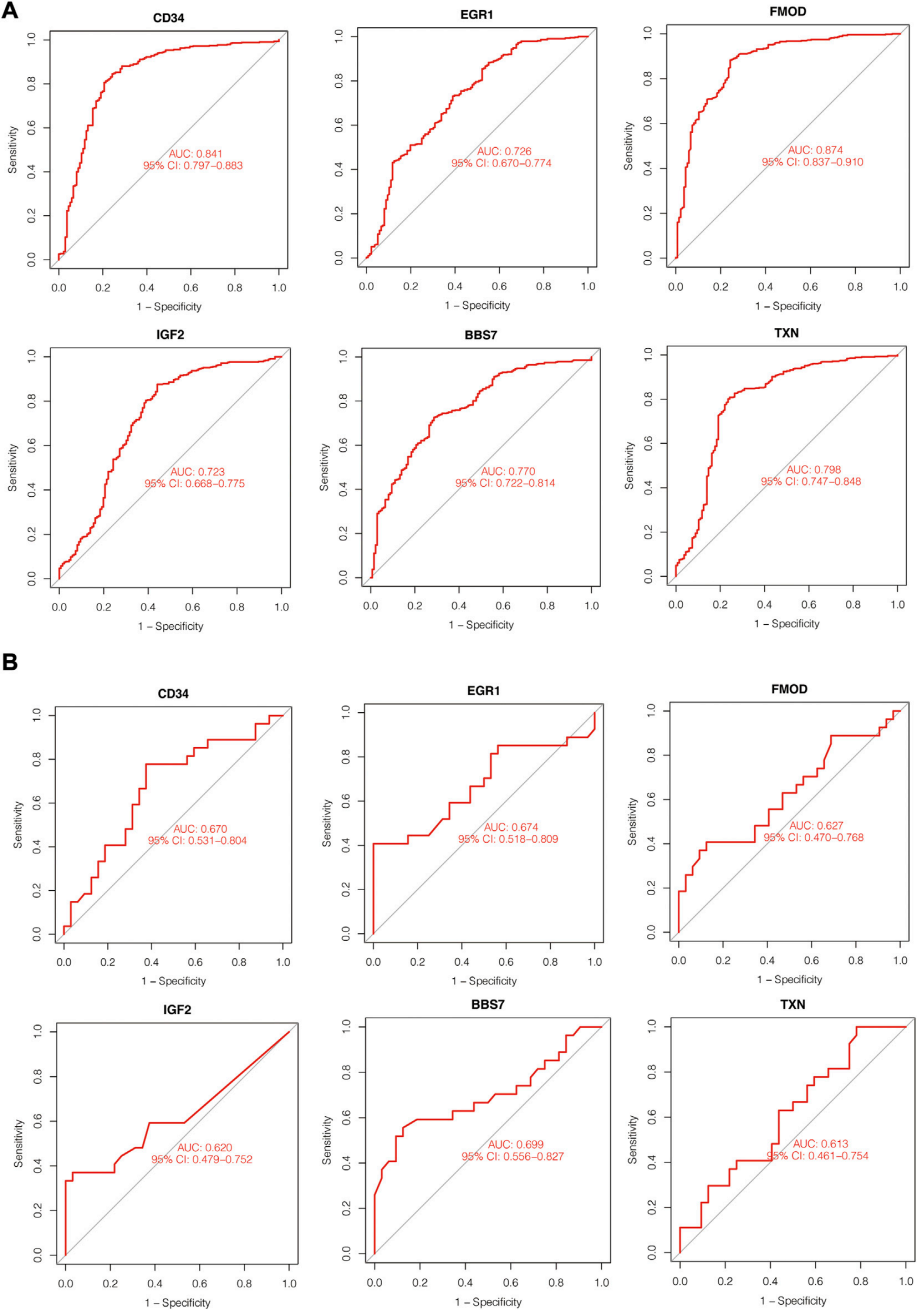
ROC curve of hub genes expression value in PD **(A)** and T1D **(B)** datasets.

To assess the diagnostic effects of hub genes, ROC analysis was performed using the abovementioned disease cohorts. The larger the area under the curve, the higher the diagnostic values. In PD-related cohort, AUC values of all the hub genes were greater than 70%, and from highest to lowest are FMOD (0.874), CD34 (0.841), TXN (0.798), BBS7 (0.77), EGR1 (0.726) and IGF2 (0.723). For T1D-related datasets, the hub genes presented less satisfactory prediction effects, and AUC for hub genes were greater than 60% overall. BBS7 (0.699) showed the highest AUC values in T1D-related datasets, followed by EGR1 (0.674), CD34 (0.67), FMOD (0.627), IGF2 (0.620) and TXN (0.613) (Figure 7).

## 4 Discussion

PD and T1D are two chronic diseases with high prevalence, and the links between the two diseases have been reported for years (Karjalainen et al., 1994; Popławska-Kita et al., 2014; Rapone et al., 2020a; Reddy and Gopalkrishna, 2022). These previous studies indicated that there may be at least one critical gene that promote the development of both PD and T1D. Nevertheless, the detailed genetic linkage is still unclear. In the era of precision medicine, discovering novel biomarkers and genetic linkage is of critical importance to treatment of the two diseases.

To make the results reliable and less biased, tissue of PD datasets and T1D datasets were from gingiva and islets separately. In this study, common DEGs of chronic PD and T1D were screened out by bioinformatics analysis. Functional enrichment analysis showed that the overlapping DEGs were mainly enriched in GO Biological Process and GO Cellular Component, including tube morphogenesis, supramolecular fiber organization, 9 + 0 non-motile cilium, plasma membrane bounded cell projection assembly, glomerulus development, enzyme-linked receptor protein signaling pathway, endochondral bone morphogenesis, positive regulation of kinase activity, cell projection membrane and regulation of lipid metabolic process. These terms are mostly about cell structure construction and cell communication. After PPI network construction and module selection, we identified 6 hub genes that are supposed to play critical roles in linking PD and T1D. Among the 6 hub genes, CD34, EGR1, FMOD and IGF2 were upregulated while BBS7 and TXN were downregulated in PD and T1D.

Among those upregulated genes, CD34 is a glycosylated transmembrane protein and widely expressed in human hematopoietic stem/progenitor cells and circulating endothelial progenitor cells (EPCs) (Zola et al., 2007; FadiniPaolo et al., 2010). Both PD and diabetes are closely related with endothelial dysfunction, and circulating EPCs is one of the endothelial dysfunction biomarkers (Gurav, 2014; Jönsson et al., 2014). It is reported that PD patients had higher count of CD34^+^ EPCs, which could be reduced by treatment of PD (Aimetti et al., 2008; Li et al., 2009; Li et al., 2011; Jönsson et al., 2014). Compared with systemically-healthy individuals with PD, gingival tissue of patients with diabetes-related PD presented more CD34^+^ cells (Penmetsa et al., 2014/11). Moreover, periodontist patient with T1D had more CD34^+^ endothelia cell counts than periodontist patients with T2D (Aspriello et al., 2009). However, a recent study showed that CD34^+^ EPCs was downregulated in PD patients. The inconsistency can be attributed to lack of specificity of CD34 as the marker of different subtypes of EPCs (Zhou et al., 2022). Different subtypes of CD34^+^ EPCs have different lifespan and physiological functions. Therefore, it may be less appropriate to use CD34 as an independent diagnostic marker and therapeutic target. CD34 is also widely used to mark the endothelial cells of blood vessels and evaluate vascular proliferation (Mirbod et al., 2001). Compared with healthy individuals, chronic PD patients with or without diabetes had more CD34 positive blood vessels in gingival tissues (Penmetsa et al., 2015). Although CD34 showed ideal diagnostic performance in this present bioinformatics study, given to the widespread presence of CD34, detailed role of CD34 in genetic linkage between PD and T1D needs more in-depth investigation.

Early growth response factor 1 (EGR1) is a zinc finger transcription factor (TF) and can be induced by a series of stimulus like shear stress and hypoxia (Silverman and Collins, 1999). EGR1 has been reported to play an important role in regulating cell proliferation, differentiation, and apoptosis (Thiel and Cibelli, 2002). Moreover, it is closely related with inflammatory process, which is a critical pathogenic mechanism of both PD and T1D (Trizzino et al., 2021/1). The relationship between EGR1 and T1D is barely reported, and the existing literature concentrate mostly on T2D. EGR1 can be induced by glucose and highly expressed in T2D Zucker Fatty rats (Josefsen et al., 1999; Garnett et al., 2005). Considering different pathogenesis of T1D and T2D, detailed role of EGR1 on T1D needs further exploration.

EGR1 is reported to be harmful for several diabetic complications, including PD, and suppressing EGR1 was helpful to alleviate the complications (Wang et al., 2015; Ao et al., 2019/5; Zha et al., 2019; Cui et al., 2022; Hu et al., 2018/). EGR1 was predicted to be involved in the regulatory network of the DEG-TFs of PD, and a bioinformatics study indicated that EGR1 may play a role in the development of PD *via* forming immunosuppressive microenvironment (Li et al., 2018; He et al., 2021). Two independent studies observed elevated expression of EGR1 in gingival fibroblasts and gingiva bulk tissue from patients with PD (Trabandt et al., 1992/5; Ebersole et al., 2018). Periodontal infection could upregulate *EGR1* expression in endothelial and Epithelial cells (Maekawa et al., 2010; Umeda et al., 2012). Macrophages, essential components of the innate immune system, have been identified to play a pivotal role in the development PD and diabetes (Zhang et al., 2021). Further study revealed that *EGR1* was upregulated in macrophages of gingival tissue with PD (Agrafioti et al., 2022). Downregulating EGR1 could suppressed inflammatory responses and M1 polarization of macrophages, which is the pro-inflammatory phenotype of macrophages and accumulated in PD and diabetes (Zhang et al., 2021; Zhi et al., 2022). In addition, tumor necrosis factor-α (TNF-α) is a pro-inflammatory cytokine identified as a pathogenic factor in both PD and DM (Nishimura et al., 2003). Studies showed that EGR1 was involved in lipopolysaccharide-induced TNF-α upregulation in macrophages (Shi et al., 2002).

Fibromodulin (FMOD) is a small leucine-rich proteoglycans (SLRPs) that can be found in teeth and bones, and normal levels of FMOD is essential for development of dental tissues and alveolar bone (Ho et al., 2013; Al-QattanMohammad and Al-Qattan, 2018). FMOD is well known for assembling the extracellular matrix and modulating collagen fibrillogenesis (Chen and Birk, 2013). It has been reported that SLRPs were crucial for periodontal homeostasis through regulating matrix turnover and collagen organization (Leong et al., 2012; Wang et al., 2014). Consistent with the present study, FMOD expression was upregulated in inflamed gingival tissue (Qian et al., 2004). It is reported that increased FMOD would be desirable for management of periodontal disease as decreased FMOD could promote osteoclastogenesis (Al-QattanMohammad and Al-Qattan, 2018). Although relationship between FMOD and T1D has not been reported, abnormal FMOD levels were observed in several diabetic complications. For instance, FMOD was identified as a potential diagnostic marker for diabetic nephropathy, and FMOD treatment can alleviate the albuminuria in diabetic nephropathy rats (Feng et al., 2021; Jazi et al., 2016/3). Considering that FMOD was implicated in inflammatory disease and cellular immune response, the detailed role of FMOD on T1D and PD deserves further exploration (Zeng-Brouwers et al., 2020).

Insulin-like growth factor 2 (IGF2) is one of the dominant members of IGFs family and is supposed to be involved in glucose metabolism (Uchimura et al., 2017). Expression level of IGF2 in T1D is tissue-specific. For instance, IGF2 expression is defective in tissues like thymus and serum, while the current study indicated that IGF2 was upregulated in islet of T1D patients (Kecha-Kamoun et al., 2001; Geenen et al., 2005; Shapiro et al., 2020). Existing literature indicated that IGF2 was a protective factor for T1D. IGF2 is critical for pancreatic β-cell mass and function and could promote proliferation of β-cells (Modi et al., 2015). Upregulating the expression of IGF2 could protect β-cells against apoptosis and deficiency of IGF2 could cause β-cell anomaly (Calderari et al., 2007; Cornu et al., 2009; Estil les et al., 2009). In addition, as an anti-apoptotic endocrine protein, IGF2 could improve survival of islet transplantation (Hughes et al., 2014). For PD, a bioinformatics study indicated that IGF2 was intersected between PD and Major depressive disorder (Sun et al., 2021). Another study also predicted that IGF2 was involved in genetic crosstalk between PD and Down Syndrome, indicating the importance of IGF2 in the pathogenesis of PD (Chen et al., 2021). IGF2 could suppress the proliferation and osteogenic differentiation of periodontal ligament cells (Konermann et al., 2013). However, detailed role of IGF2 on PD still needs further experimental validation.

Bardet-Biedl syndrome (BBS) is an autosomal recessive disorder characterized by a series of anomalies like obesity, hypertension and diabetes (Zhang et al., 2012). As one of the disease-causing gene, BBS7 is involved in encoding BBSome complex, which plays a critical role in primary cilia function and intracellular transport (Zhang et al., 2013). Although diabetes is one of the common features of Bardet-Biedl syndrome, the detailed relationship between BBS7 and diabetes remains unclear. For PD, BBS7 is essential for periodontal ligament homeostasis through regulating primary cilia. BBS7 was downregulated in occlusal hypofunctional periodontal ligament, and knockdown of BBS7 could inhibit cell migration and angiogenesis (Chang et al., 2021).

It is reported that thioredoxin (TXN) could protect cells from oxidative stress, which is among causes of destruction of pancreatic β-cells in T1D (Kaneto et al., 2007). Overexpression of TXN in pancreatic β-cells could reduce the incidence of T1D (Hotta et al., 1998). In addition, *TXN* polymorphism can influence the susceptibility of T1D (Ikegami et al., 2008). However, the relationship between TXN and PD has not been reported yet.

Through analyzing these hub genes, we found that although the expression trends of these hub genes were the same in PD and T1D datasets, their effects on the disease progression can be opposite. This phenomenon further suggests the importance of precision therapy and the “precision” is reflected not only in key biomarkers, but also in targeted tissue.

However, several limitations did exist in this study. The transcriptome data comes from different population, causing bias to some extent. In addition, imbalanced sample size of PD and T1D cohorts may lead to a shift in detected genes. Therefore, to overcome this limitations, further experimental validation is needed.

## 5 Conclusion

In the present study, we uncovered several biological processes and pathways that the DEGs were enriched in *via* bioinformatic analysis. Six key genes were identified and validated that may play a pivotal role in crosstalk between PD and T1D. Some of them have been implicated to be involved in PD or T1D, showing great potential to be key targets for diagnosis and treatments. Our findings provide novel insights into crosstalk between PD and T1D and pave the way for scientific researches and the development of therapeutic strategies. Targeting at the common expression genes may be a potential strategy to tackle both diseases at the same time. In the future, *in vitro* and *in vivo* validations are expected to further confirm our findings.

## Data Availability

The datasets presented in this study can be found in online repositories. The names of the repository/repositories and accession number (s) can be found in the article/Supplementary Material.

## Abbreviations

AUC, area under the curve; BBS, bardet-biedl syndrome; DEGs, differentially expressed genes; DM, diabetes mellitus; EPCs, endothelial progenitor cells; EGR1, early growth response factor 1; FMOD, fibromodulin; GEO, gene expression omnibus; GO, gene ontology; IGF2, insulin-like growth factor 2; KEGG, kyoto encyclopedia of genes and genomes; PD, periodontitis; PCA, principal component analysis; PPI, protein-protein interaction; ROC, receiver operating characteristic; STRING, the search tool for the retrieval of interacting genes/proteins; SLRPs, small leucine-rich proteoglycans; T1D, type 1 diabetes; T2D, type 2 diabetes; TF, transcription factor; TNF-α, tumor necrosis factor-α; TXN, thioredoxi.

## References

[B1] Abe D. Kubota T. Morozumi T. Shimizu T. Nakasone N. Itagaki M. (2011). Altered gene expression in leukocyte transendothelial migration and cell communication pathways in periodontitis-affected gingival tissues. J. periodontal Res. 46, 345–353. 10.1111/j.1600-0765.2011.01349.x 21382035

[B2] Agrafioti P. Morin-Baxter J. KranthiTanagala K. K. Dubey S. Sims P. Lalla E. (2022). Decoding the role of macrophages in periodontitis and type 2 diabetes using single-cell RNA-sequencing. FASEB J. 36 (2), e221366. 10.1096/fj.202101198R PMC8881186 35032412

[B3] Aimetti M. Romano F. Marsico A. Navone R. (2008). Non-surgical periodontal treatment of cyclosporin a-induced gingival overgrowth: Immunohistochemical results. Oral Dis. 14, 244–250. 10.1111/j.1601-0825.2007.01364.x 18266838

[B4] Al-Qattan M. M. Al-Qattan A. M. (2018). Fibromodulin: Structure, physiological functions, and an emphasis on its potential clinical applications in various diseases. J. Coll. Physicians Surgeons--Pakistan JCPSP 28, 783–790. 30266125

[B5] Ao H. Liu B. Li H. Lu L. (2019). Egr1 mediates retinal vascular dysfunction in diabetes mellitus via promoting P53 transcription. J. Cell. Mol. Med. 23 (5), 3345–3356. 10.1111/jcmm.14225 30887692 PMC6484413

[B6] Aren G. Sepet E. Ozdemir D. Dinççağ N. Güvener B. Firatli E. (2003). Periodontal health, salivary status, and metabolic control in children with type 1 diabetes mellitus. J. Periodontol. 74, 1789–1795. 10.1902/jop.2003.74.12.1789 14974821

[B7] Aspriello S. D. Zizzi A. Lucarini G. Rubini C. Faloia E. Boscaro M. (2009). Vascular endothelial growth factor and microvessel density in periodontitis patients with and without diabetes. J. periodontology 80, 1783–1789. 10.1902/jop.2009.090239 19905947

[B8] Barrett T. Wilhite S. E. Ledoux P. Evangelista C. Kim I. F. Tomashevsky M. (2013). Ncbi geo: Archive for functional Genomics data sets--update. Nucleic acids Res. 41, D991–D995. Database issue. 10.1093/nar/gks1193 23193258 PMC3531084

[B9] Calderari S. Gangnerau M. N. Thibault M. Meile M. J. Kassis N. Alvarez C. (2007). Defective Igf2 and Igf1r protein production in embryonic pancreas precedes beta cell mass anomaly in the goto-kakizaki rat model of type 2 diabetes. Diabetologia 50, 1463–1471. 10.1007/s00125-007-0676-2 17476475

[B10] Chang P. E. Li S. Kim H. Y. Lee D. J. Choi Y. J. Jung H. S. (2021). BBS7-SHH signaling activity regulates primary cilia for periodontal homeostasis. Front. Cell Dev. Biol. 9, 796274. 10.3389/fcell.2021.796274 34957122 PMC8703258

[B11] Chen S. Birk D. E. (2013). The regulatory roles of small leucine-rich proteoglycans in extracellular matrix assembly. FEBS J. 280, 2120–2137. 10.1111/febs.12136 23331954 PMC3651807

[B12] Chen Y. Yu X. Kong J. (2021). Identification of neuropeptides as potential crosstalks linking Down syndrome and periodontitis revealed by transcriptomic analyses. Dis. Markers 2021, 7331821. 10.1155/2021/7331821 34545294 PMC8449741

[B13] Chin C-H. Chen S-H. Wu H-H. Ho C-W. Ko M-T. Lin C-Y. (2014). Cytohubba: Identifying hub objects and sub-networks from complex inter actome. BMC Syst. Biol. 8 (4), S11. 10.1186/1752-0509-8-S4-S11 25521941 PMC4290687

[B14] Corbella S. Francetti L. Taschieri S. De Siena F. Fabbro M. D. (2013). Effect of periodontal treatment on glycemic control of patients with diabetes: A systematic review and meta-analysis. J. Diabetes Investig. 4, 502–509. 5 (Sep 13 2013). 10.1111/jdi.12088 PMC4025114 24843701

[B15] Cornu M. Yang J. Y. Jaccard E. Poussin C. Widmann C. Thorens B. (2009). Glucagon-like peptide-1 protects beta-cells against apoptosis by increasing the activity of an IGF-2/IGF-1 receptor autocrine loop. Diabetes 58, 1816–1825. 10.2337/db09-0063 19401425 PMC2712796

[B16] Cui K-M. Hu Z-P. Wang Y. L. (2022). Mg53 represses high glucose-induced inflammation and angiogenesis in human retinal endothelial cells by repressing the egr1/stat3 Axis. Immunopharmacol. Immunotoxicol. 44, 484–491. 10.1080/08923973.2022.2054426 35438597

[B17] Dakovic D. Pavlovic M. D. (2008). Periodontal disease in children and adolescents with type 1 diabetes in Serbia. J. Periodontol. 79 (6), 987–992. 10.1902/jop.2008.070549 18533774

[B18] Demmer R. T. Behle J. H. Wolf D. L. Martin H. Kebschull M. Celenti R. (2008). Transcriptomes in healthy and diseased gingival tissues. J. periodontology 79, 2112–2124. 10.1902/jop.2008.080139 PMC2637651 18980520

[B19] Dicembrini I. Serni L. Monami M. Caliri M. Barbato L. Cairo F. (2020). Type 1 diabetes and periodontitis: Prevalence and periodontal destruction-a systematic review. Acta Diabetol. 57, 1405–1412. 10.1007/s00592-020-01531-7 32318875

[B20] Ebersole J. L. Michael John Novak L. O. Martinez-Gonzalez J. Kirakodu S. Chen K. C. Arnold S. (2018). Hypoxia-inducible transcription factors, Hif1a and Hif2a, increase in aging mucosal tissues. Immunology 154, 452–464. 10.1111/imm.12894 29338076 PMC6002220

[B21] Eke P. I. Borgnakke W. S. Genco R. J. (2000). Recent epidemiologic trends in periodontitis in the USA [in eng]. Periodontol 82, 257–267. 10.1111/prd.12323 31850640

[B22] Emrich L. J. Shlossman M. Genco R. J. (1991). Periodontal disease in non-insulin-dependent diabetes mellitus. J. periodontology 62, 123–131. 10.1902/jop.1991.62.2.123 2027060

[B23] Engebretson S. Kocher T. (2013). Evidence that periodontal treatment improves diabetes outcomes: A systematic review and meta-analysis. J. periodontology 84 (4), S153–S169. 10.1902/jop.2013.1340017 PMC4100543 23631575

[B24] Estil les E. Téllez N. Soler J. Montanya E. (2009). High sensitivity of beta-cell replication to the inhibitory effects of interleukin-1beta: Modulation by adenoviral overexpression of IGF2 in rat islets. J. Endocrinol. 203, 55–63. 10.1677/joe-09-0047 19592596

[B25] Fadini G. P. Pagano C. Baesso I. Kotsafti O. Doro D. De Kreutzenberg S. V. (2010). Reduced endothelial progenitor cells and brachial artery flow-mediated dilation as evidence of endothelial dysfunction in ocular hypertension and primary open-angle glaucoma. Acta Ophthalmol. 88, 135–141. 10.1111/j.1755-3768.2009.01573.x 19549102

[B26] Feng S. Gao Y. Yin D. Lv L. Wen Y. Li Z. (2021). Identification of lumican and fibromodulin as hub genes associated with accumulation of extracellular matrix in diabetic nephropathy. Kidney & blood Press. Res. 46 (3), 275–285. 10.1159/000514013 33887734

[B27] Garnett K. E. Philip Chapman J. A. C. I. D. W. Boam D. S. W. Waddell I. D. (2005). Differential gene expression between zucker fatty rats and zucker diab etic fatty rats: A potential role for the immediate-early gene egr-1 in regulation of beta cell proliferation. J. Mol. Endocrinol. 35, 13–25. 10.1677/jme.1.01792 16087718

[B28] Geenen V. Brilot F. Louis C. Hansenne I. Renard C. Martens H. (2005). Importance of a thymus dysfunction in the pathophysiology of type 1 diabetes. Rev. medicale Liege 60 (5-6), 291–296. 16035283

[B29] Graziani F. Gennai S. Anna S. Petrini M. (2018). "A systematic review and meta-analysis of epidemiologic observational evidence on the effect of periodontitis on diabetes an update of the EFP-AAP review." J. Clin. periodontology 45, 167–187. 10.1111/jcpe.12837 29277926

[B30] Gurav A. N. (2014). The implication of periodontitis in vascular endothelial dysfunction. Eur. J. Clin. investigation 44, 1000–1009. 10.1111/eci.12322 25104241

[B31] He L. Liu L. Li T. Zhuang D. Dai J. Wang B. (2021). Exploring the imbalance of periodontitis immune system from the cellular to molecular level. Front. Genet. 12, 653209. 10.3389/fgene.2021.653209 33841510 PMC8033214

[B32] Ho S. P. Kurylo M. P. Grandfield K. Hurng J. Herber R. P. Ryder M. I. (2013). The plastic nature of the human bone-periodontal ligament-tooth fibrous joint. Bone 57, 455–467. 10.1016/j.bone.2013.09.007 24063947 PMC3938967

[B33] Hodge P. J. Robertson D. Paterson K. Smith G. L. Creanor S. Sherriff A. (2012). Periodontitis in non-smoking type 1 diabetic adults: A cross-sectional study. J. Clin. Periodontol. 39, 20–29. 10.1111/j.1600-051X.2011.01791.x 22092931

[B34] Hotta M. Tashiro F. Ikegami H. Niwa H. Ogihara T. Yodoi J. (1998). Pancreatic beta cell-specific expression of thioredoxin, an antioxidative and antiapoptotic protein, prevents autoimmune and streptozotocin-induced diabetes. J. Exp. Med. 188 (8), 1445–1451. 10.1084/jem.188.8.1445 9782121 PMC2213419

[B35] Hu F. Xue M. Yang L. Jia Y-J. Zheng Z-J. Yang Y-L. (2018). Early growth response 1 (Egr1) is a transcriptional activator of Nox4 in oxidative stress of diabetic kidney disease. J. diabetes Res. 2018, 3405695. 10.1155/2018/3405695 29854821 PMC5944279

[B36] Hughes A. Rojas-Canales D. Drogemuller C. Voelcker N. H. Grey S. T. Coates P. T. (2014). IGF2: An endocrine hormone to improve islet transplant survival. J. Endocrinol. 221, R41–R48. 10.1530/joe-13-0557 24883437

[B37] Ikegami H. Ono M. Fujisawa T. Hiromine Y. Kawabata Y. Yamato E. (2008). Molecular scanning of the gene for thioredoxin, an antioxidative and antiapoptotic protein, and genetic susceptibility to type 1 diabetes. Ann. N. Y. Acad. Sci. 1150, 103–105. 10.1196/annals.1447.060 19120277

[B38] Ismail A. F. McGrath C. P. Yiu C. K. (2015). Oral health of children with type 1 diabetes mellitus: A systematic review. Diabetes Res. Clin. Pract. 108 (3), 369–381. 10.1016/j.diabres.2015.03.003 25817182

[B39] Janket S. J. Wightman A. Baird A. E. Van Dyke T. E. Jones J. A. (2005). Does periodontal treatment improve glycemic control in diabetic patients? A meta-analysis of intervention studies. J. Dent. Res. 84, 1154–1159. 10.1177/154405910508401212 16304446 PMC1797067

[B40] Jazi M. F. Biglari A. Mazloomzadeh S. Paul K. Ali R. Eskandari M. (2016). Recombinant fibromodulin has therapeutic effects on diabetic nephropathy by down-regulating transforming growth factor-ß1 in streptozotocin- induced diabetic rat model. Iran. J. basic Med. Sci. 19 (3), 265–271. 27114796 PMC4834116

[B41] Jindal A. Parihar A. S. Sood M. Singh P. Singh N. (2015). Relationship between severity of periodontal disease and control of diabetes (glycated hemoglobin) in patients with type 1 diabetes mellitus. J. Int. Oral Health 7 (2), 17–20. PMC4672852 26668475

[B42] Jönsson D. Spinell T. Vrettos A. Stoecklin-Wasmer C. Celenti R. Demmer R. T. (2014). Circulating endothelial progenitor cells in periodontitis. J. periodontology 85, 1739–1747. 10.1902/jop.2014.140153 25101916

[B43] Josefsen K. Sørensen L. R. Buschard K. Birkenbach M. (1999). Glucose induces early growth response gene (Egr-1) expression in pancreatic beta cells. Diabetologia 42, 195–203. 10.1007/s001250051139 10064100

[B44] Kakleas K. Soldatou A. Karachaliou F. Karavanaki K. (2015). Associated autoimmune diseases in children and adolescents with type 1 diabetes mellitus (T1dm). Autoimmun. Rev. 14, 81–97. 10.1016/j.autrev.2015.05.002 26001590

[B45] Kaneto H. Matsuoka T. A. Katakami N. Kawamori D. Miyatsuka T. Yoshiuchi K. (2007). Oxidative stress and the JNK pathway are involved in the development of type 1 and type 2 diabetes. Curr. Mol. Med. 7 (7), 674–686. 10.2174/156652407782564408 18045145

[B46] Karjalainen K. M. Knuuttila M. L. von Dickhoff K. J. (1994). Association of the severity of periodontal disease with organ complications in type 1 diabetic patients. J. Periodontol. 65, 1067–1072. 10.1902/jop.1994.65.11.1067 7853131

[B47] Kebschull M. Demmer R. T. Grün B. Guarnieri P. Pavlidis P. Papapanou P. N. (2014). Gingival tissue transcriptomes identify distinct periodontitis phenotypes. J. Dent. Res. 93 (5), 459–468. 10.1177/0022034514527288 24646639 PMC3988627

[B48] Kecha-Kamoun O. Achour I. Martens H. Collette J. Lefebvre P. J. Greiner D. L. (2001). Thymic expression of insulin-related genes in an animal model of autoimmune type 1 diabetes. Diabetes Metab. Res. Rev. 17, 146–152. 10.1002/dmrr.182 11307180

[B49] Konermann A. Lossdörfer S. Jäger A. Chen Y. Götz W. (2013). Autoregulation of insulin-like growth factor 2 and insulin-like growth factor-binding protein 6 in periodontal ligament cells *in vitro* . Ann. Anat. 195, 527–532. 10.1016/j.aanat.2013.10.001 24182837

[B50] Lalla E. Cheng B. Lal S. Kaplan S. Softness B. Greenberg E. (2007). Diabetes mellitus promotes periodontal destruction in children. J. Clin. periodontology 34, 294–298. 10.1111/j.1600-051X.2007.01054.x 17378885

[B51] Leong N. L. Hurng J. M. Ho S. P. Ho. S. P. (2012). Age-related adaptation of bone-PDL-tooth complex: Rattus-norvegicus as a model system. PloS one 7 (4), e35980. 10.1371/journal.pone.0035980 22558292 PMC3340399

[B52] Li S. Liu X. Li H. Pan H. Acharya A. Deng Y. (2018). Integrated analysis of long noncoding RNA-associated competing endogenous RNA network in periodontitis. J. periodontal Res. 53, 495–505. 10.1111/jre.12539 29516510

[B53] Li X. HungTse F. Hang Yiu K. Jia N. Chen H. Jin L. (2009). Increased levels of circulating endothelial progenitor cells in subjects with moderate to severe chronic periodontitis. J. Clin. periodontology 36, 933–939. 10.1111/j.1600-051X.2009.01481.x 19799717

[B54] Li X. HungTse F. Jin L. (2011). Effect of periodontal treatment on circulating Cd34(+) cells and peripheral vascular endothelial function: A randomized controlled trial. J. Clin. periodontology 38, 148–156. 10.1111/j.1600-051X.2010.01651.x 21133981

[B55] Maahs D. M. West N. A. Lawrence J. M. Mayer-Davis E. J. (2010). Epidemiology of type 1 diabetes. Endocrinol. metabolism Clin. N. Am. 39, 481–497. 10.1016/j.ecl.2010.05.011 PMC2925303 20723815

[B56] Madianos P. N. Koromantzos P. A. (2018). An update of the evidence on the potential impact of periodontal therapy on diabetes outcomes. J. Clin. periodontology 45, 188–195. 10.1111/jcpe.12836 29277978

[B57] Maekawa T. Takahashi N. Honda T. Yonezawa D. Miyashita H. Okui T. (2010). Porphyromonas gingivalis antigens and interleukin-6 stimulate the production of monocyte chemoattractant protein-1 via the upregulation of early growth response-1 transcription in human coronary artery endothelial cells. J. Vasc. Res. 47 (4), 346–354. 10.1159/000265568 20016208

[B58] Mealey B. L. Ocampo G. L. (2000). Diabetes mellitus and periodontal disease. Periodontology 44, 127–153. 10.1111/j.1600-0757.2006.00193.x 17474930

[B59] Mirbod S. M. Ahing S. I. Pruthi V. K. (2001). Immunohistochemical study of vestibular gingival blood vessel density and internal circumference in smokers and non-smokers. J. periodontology 72, 1318–1323. 10.1902/jop.2001.72.10.1318 11699472

[B60] Modi H. Jacovetti C. Tarussio D. Metref S. Madsen O. D. Zhang F. P. (2015), Autocrine action of IGF2 regulates adult β-cell mass and function. Diabetes 64, 4148–4157. 10.2337/db14-1735 26384384

[B61] Nishimura F. Iwamoto Y. Mineshiba J. Shimizu A. Soga Y. Murayama Y. (2003). Periodontal disease and diabetes mellitus: The role of tumor necrosis factor-alpha in a 2-way relationship. J. periodontology 74, 97–102. 10.1902/jop.2003.74.1.97 12593603

[B62] Novotna M. Podzimek S. Broukal Z. Lencova E. Duskova J. (2015). Periodontal diseases and dental caries in children with type 1 diabetes mellitus. Mediat. Inflamm. 2015, 379626. [In eng]. 10.1155/2015/379626 PMC4539482 26347009

[B63] Orbak R. Simsek S. Orbak Z. Kavrut F. Colak M. (2008). The influence of type-1 diabetes mellitus on dentition and oral health in children and adolescents. Yonsei Med. J. 49, 357–365. 3 (Jun 30 2008). 10.3349/ymj.2008.49.3.357 18581583 PMC2615350

[B64] Papapanou P. N. Sanz M. Buduneli N. Dietrich T. Feres M. Fine D. H. (2018). Periodontitis: Consensus report of workgroup 2 of the 2017 world works hop on the classification of periodontal and peri-implant diseases and conditions. J. periodontology 89, S173–S182. 10.1002/JPER.17-0721 29926951

[B65] Penmetsa G. S. Satyanarayana B. Manyam R. Doraswamy Dwarakanath C. (2015). Comparison of the number of gingival blood vessels between type 2 diabetes mellitus and chronic periodontitis patients: An immunohistological study. J. Indian Soc. Periodontology 19 (2), 164–168. 10.4103/0972-124X.152105 PMC4439625 26015666

[B66] Penmetsa G. S. Mandalapu N. Dv S. Mannem R. Alla R. K. Gadde P. (2014). Immunolocalization of CD34 positive progenitor cells in diabetic and non diabetic periodontitis patients - a comparative study. J. Clin. diagnostic Res. JCDR 8, ZC96–ZC99. ZC96-9. 10.7860/JCDR/2014/9827.5191 PMC4290284 25584328

[B67] Plessas A. Robertson D. P. Hodge P. J. (2018). Radiographic bone loss in a scottish non-smoking type 1 diabetes mellitus population: A bitewing radiographic study. J. Periodontol. 89, 1043–1051. 10.1002/jper.16-0788 29766516

[B68] Popławska-Kita A. Siewko K. Szpak P. Król B. Telejko B. Klimiuk P. A. (2014). Association between type 1 diabetes and periodontal health. Adv. Med. Sci. 59 (1), 126–131. 10.1016/j.advms.2014.01.002 24797988

[B69] Pranckeviciene A. Siudikiene J. Ostrauskas R. Machiulskiene V. (2017). Long-term effect of periodontal surgery on oral health and metabolic control of diabetics. Clin. Oral Investig. 21 (3), 735–743. 10.1007/s00784-016-1819-y 27068410

[B70] Preshaw P. M. Alba A. L. Herrera D. Jepsen S. Konstantinidis A. Makrilakis K. (2012). "Periodontitis and diabetes: A two-way relationship. Diabetologia 55, 21–31. 10.1007/s00125-011-2342-y 22057194 PMC3228943

[B71] Pujar M. Vastrad B. Kavatagimath S. Vastrad C. Kotturshetti S. (2022). Identification of candidate biomarkers and pathways associated with type 1 diabetes mellitus using bioinformatics analysis. Sci. Rep. 12, 9157. 10.1038/s41598-022-13291-1 35650387 PMC9160069

[B72] Qian H. Xiao Y. Bartold P. M. (2004). Immunohistochemical localization and expression of fibromodulin in adult rat periodontium and inflamed human gingiva. Oral Dis. 10, 233–239. 10.1111/j.1601-0825.2004.00996.x 15196146

[B73] Rapone B. Corsalini M. Converti I. Loverro M. T. Gnoni A. Trerotoli P. (2020). Does periodontal inflammation affect type 1 diabetes in childhood and adolescence? A meta-analysis [in eng]. Front. Endocrinol. (Lausanne) 11, 278. 10.3389/fendo.2020.00278 32431669 PMC7214631

[B74] Rapone B. Ferrara E. Corsalini M. Converti I. Grassi F. R. Santacroce L. (2020). The effect of gaseous ozone therapy in conjunction with periodontal treatment on glycated hemoglobin level in subjects with type 2 diabetes mellitus: An unmasked randomized controlled trial. Int J Environ Res Public Health 17 (15), 5467. 10.3390/ijerph17155467 32751340 PMC7432743

[B75] Rayburn W. F. (1997). Diagnosis and classification of diabetes mellitus: Highlights from the American diabetes association. J. reproductive Med. 42 (9), 585–586. 9336756

[B76] Reddy M. Gopalkrishna P. (2022). Type 1 diabetes and periodontal disease: A literature review. Can. J. Dent. Hyg. CJDH = J. Can. de l'hygi ene Dent. JCHD 56, 22–30. PMC8937570 35401764

[B77] Roy M. Gastaldi G. Courvoisier D. S. Mombelli A. Giannopoulou C. (2019). Periodontal health in a cohort of subjects with type 1 diabetes mellitus. Clin. Exp. Dent. Res. 5 (3), 243–249. 10.1002/cre2.178 31249705 PMC6585577

[B78] Seiron P., Stenwall A. Anders H. Granlund L. Esguerra J. L. S. Volkov P. (2021). Transcriptional analysis of islets of langerhans from organ donors of different ages. PloS one 16 (3), e0247888. 10.1371/journal.pone.0247888 33711030 PMC7954335

[B79] Shapiro M. R. Wasserfall C. H. McGrail S. M. Posgai A. L. Bacher R. Muir A. (2020). Insulin-like growth factor dysregulation both preceding and following type 1 diabetes diagnosis. Diabetes 69, 413–423. 10.2337/db19-0942 31826866 PMC7034187

[B80] Shi L. Kishore R. McMullen M. R. Nagy L. E. (2002). Lipopolysaccharide stimulation of erk1/2 increases tnf-alpha production via egr-1. Am. J. physiology. Cell physiology 282–C1211. 10.1152/ajpcell.00511.2001 11997234

[B81] Shinjo T. Ishikado A. Hasturk H. Pober D. M. Paniagua S. M. Shah H. (2019). Characterization of periodontitis in people with type 1 diabetes of 50 years or longer duration. J. Periodontol. 90 (6), 565–575. 10.1002/jper.18-0735 31026349 PMC7087383

[B82] Silverman E. S. Collins T. (1999). Pathways of EGR-1-mediated gene transcription in vascular biology. Am. J. pathology 154, 665–670. 10.1016/S0002-9440(10)65312-6 PMC1866415 10079243

[B83] Simeni Njonnou S. R. Boombhi J. Etoa Etoga M. C. Tiodoung Timnou A. Jingi A. M. Nkem Efon K. (2020). Prevalence of diabetes and associated risk factors among a group of prisoners in the yaoundé central prison. J. Diabetes Res. 2020, 5016327. 10.1155/2020/5016327 32047824 PMC7003275

[B84] Sun C. Han J. Bai Y. Zhong Z. Song Y. Sun Y. (2021). Neuropeptides as the shared genetic crosstalks linking periodontitis and major depression disorder. Dis. 2021, 3683189. 10.1155/2021/3683189 PMC8553477 34721734

[B85] Sundar C. Ramalingam S. Mohan V. Pradeepa R. Ramakrishnan M. J. (2018). Periodontal therapy as an adjunctive modality for Hba1c reduction in type-2 diabetic patients. J. Educ. health Promot. 7, 152. 10.4103/jehp.jehp_66_18 30788374 PMC6333026

[B86] Szklarczyk D. Gable A. L. Lyon D. Junge A. Wyder S. Huerta-Cepas J. (2019). String V11: Protein-protein association networks with increased coverage, supporting functional discovery in genome-wide experimental datase ts. Nucleic acids Res. 47, D607–D13. 10.1093/nar/gky1131 30476243 PMC6323986

[B87] Taylor J. J. Preshaw P. M. Evanthia L. (2013). A review of the evidence for pathogenic mechanisms that may link periodontitis and diabetes. J. periodontology 84 (4), S113–S134. 10.1902/jop.2013.134005 23631573

[B88] Thiel G. Cibelli G. (2002). Regulation of life and death by the zinc finger transcription factor EGR-1. J. Cell. physiology 193 (3), 287–292. 10.1002/jcp.10178 12384981

[B89] Trabandt A. Gay R. E. Sukhatme V. P. Gay S. (1992). Expression of collagenase and potential transcriptional factors C-fos and egr-1 in periodontal gingival fibroblasts. J. oral pathology Med. official Publ. Int ernational Assoc. Oral Pathologists Am. Acad. o f Oral Pathology 21 (5), 232–240. 10.1111/j.1600-0714.1992.tb00108.x 1383501

[B90] Trizzino M. Avery Z. Deliard S. Wang F. Barbieri E. Veglia F. (2021). EGR1 is a gatekeeper of inflammatory enhancers in human macrophages. Sci. Adv. 7, eaaz8836–3. eaaz8836. 10.1126/sciadv.aaz8836 33523892 PMC7806227

[B91] Tsai C. Hayes C. Taylor G. W. (2002). Glycemic control of type 2 diabetes and severe periodontal disease in the us adult population. Community Dent. oral Epidemiol. 30, 182–192. 10.1034/j.1600-0528.2002.300304.x 12000341

[B92] Uchimura T. Hollander J. M. Nakamura D. S. Liu Z. Rosen C. J. Georgakoudi I. (2017). An essential role for Igf2 in cartilage development and glucose metabolism during postnatal long bone growth. Dev. Camb. Engl. 144, 3533–3546. 10.1242/dev.155598 PMC5665487 28974642

[B93] Umeda J. E. Demuth D. R. Ando E. S. Faveri M. Mayer M. P. A. (2012). Signaling transduction analysis in gingival epithelial cells after infection with aggregatibacter actinomycetemcomitans. Mol. oral Microbiol. 27, 23–33. 10.1111/j.2041-1014.2011.00629.x 22230463 PMC3257577

[B94] Wang D. Guan M-P. Zheng Z-J. Li W-Q. Lyv F-P. Pang R-Y. (2015). Transcription factor Egr1 is involved in high glucose-induced proliferation and fibrosis in rat glomerular mesangial cells. Cell. physiology Biochem. Int. J. experi Ment. Cell. physiology, Biochem. Pharmacol. 36 (6), 2093–2107. 10.1159/000430177 26279418

[B95] Wang L. Foster B. L. Kram V. Nociti F. H. Jr. Zerfas P. M. Tran A. B. (2014). Fibromodulin and biglycan modulate periodontium through tgfβ/bmp signaling. J. Dent. Res. 93, 780–787. 10.1177/0022034514541126 24966230 PMC4126226

[B96] Zeng-Brouwers J. Pandey S. Trebicka J. Wygrecka M. Schaefer L. (2020). Communications via the small leucine-rich proteoglycans: Molecular specificity in inflammation and autoimmune diseases. J. Histochem. Cytochem. official J. Histochem. Soc. 68, 887–906. 10.1369/0022155420930303 PMC7708667 32623933

[B97] Zha F. Qu X. Tang B. Li J. Wang Y. Zheng P. (2019). Long non-coding RNA Meg3 promotes fibrosis and inflammatory response in diabetic nephropathy via MiR-181a/egr-1/tlr4 Axis. Aging 11, 3716–3730. 10.18632/aging.102011 31195367 PMC6594792

[B98] Zhang B. Yang Y. Yi J. Zhao Z. Ye R. (2021). Hyperglycemia modulates M1/M2 macrophage polarization via reactive oxygen species overproduction in ligature-induced periodontitis. J. periodontal Res. 56, 991–1005. 10.1111/jre.12912 34190354

[B99] Zhang Q. Nishimura D. Vogel T. Shao J. Swiderski R. Yin T. (2013). BBS7 is required for BBSome formation and its absence in mice results in Bardet-Biedl syndrome phenotypes and selective abnormalities in membrane protein trafficking. J. Cell Sci. 126, 2372–2380. Pt 11 (Jun 1 2013). 10.1242/jcs.111740 23572516 PMC3679484

[B100] Zhang Q. Seo S. Bugge K. Stone E. M. Sheffield V. C. (2012). BBS proteins interact genetically with the IFT pathway to influence SHH-related phenotypes. Hum. Mol. Genet. 21 (9), 1945–1953. 10.1093/hmg/dds004 22228099 PMC3315203

[B101] Zhi Y-K. Jing L. Lang Y. Zhu R-L. Luo J-F. Shi Q-P. (2022). Sinomenine inhibits macrophage M1 polarization by downregulating α7nAChR via a feedback pathway of α7nAChR/ERK/Egr-1. Phytomedicine Int. J. phytotherapy Phytopharm. ology 100, 154050. 10.1016/j.phymed.2022.154050 35397284

[B102] Zhou J. Chen S. Ren J. Zou H. Liu Y. Chen Y. (2022). Association of enhanced circulating trimethylamine N-oxide with vascular endothelial dysfunction in periodontitis patients. J. periodontology 93 (5), 770–779. 10.1002/JPER.21-0159 34472093

[B103] Zhou Y. Zhou B. Pache L. Chang M. Tanaseichuk O. Benner C. (2019). Metascape provides a biologist-oriented resource for the analysis of systems-level datasets. Nat. Commun. 10 (1), 1523. 10.1038/s41467-019-09234-6 30944313 PMC6447622

[B104] Zola H. Swart B. Banham A. Barry S. Beare A. Armand B. (2007). CD molecules 2006--human cell differentiation molecules. J. Immunol. methods 319, 1–5. 10.1016/j.jim.2006.11.001 17174972

